# A new method for estimating time since death by analysis of substances deposited on the surface of dental enamel in a body immersed in seawater

**DOI:** 10.1007/s00414-019-02020-5

**Published:** 2019-02-15

**Authors:** Noboru Ishikawa, Yasuo Miake, Kei Kitamura, Hitoshi Yamamoto

**Affiliations:** 1 Department of Histology and Developmental Biology, Tokyo Dental College, 2-9-18 Kanda-Misakicho, Chiyoda, Tokyo, 101-0061 Japan; 2 Department of Forensic Medicine, Kyoto Prefectural University of Medicine, Kyoto, 602-8566 Japan

**Keywords:** Forensic odontology, Time after death, Seawater, Immersed body, Dental enamel, Drowning

## Abstract

The present investigation was performed with the objective of developing a method to estimate how long a corpse had been immersed in water after death (the time since death). Accurate determination of the time elapsed since death may lead to identification of the place of drowning, and therefore, serves not only as a piece of information useful for determination of the cause of death but also leads to prompt identification of the body. The results showed that diatoms attached to the surface of dental enamel increased with prolongation of immersion time in water. Further, as the immersion time increased, the quantity of O, Si, Mg, K, Al, and S detected on the surface of dental enamel increased, while the quantity of the main dental components (Ca and P) that were detected gradually decreased. Based on these results, we calculated a regression formula to estimate the immersion time. Our method is considered to be a breakthrough technique for evaluating the time since death more objectively, compared to the conventional method of determination based on the degree of decomposition of the corpse.

## Introduction

Estimation of the time of death is one of the main tasks in forensic medicine. Numerous reports have been published concerning methods used for estimating the time since death [1–12]. However, as most studies have mainly examined soft tissues, these findings cannot be applied easily for severely decomposed corpses. Estimation of the time of death is often difficult, especially for corpses that are discovered outdoors. Possible reasons for this are strong influence of the site at which the corpse was discovered (aboveground, underwater, or underground), season (temperature, humidity), and extent of damage done by animals or insects. Particularly, when the corpse remains underwater or underground, it often takes a long time to find the body after death, and thus, influence of the environment is even stronger. Unlike corpses that are discovered aboveground or underground, the place of death often differs from the place of discovery in case of corpses that are discovered underwater. Accordingly, accurate determination of the time since death may lead to identification of the place of drowning; therefore, it not only serves as a piece of information that is useful for determining the cause of death but also leads to prompt identification of the body. In forensic medicine, the degree of adipocere as well as general postmortem phenomena is taken into consideration while estimating the time since death in case of underwater corpses [13, 14]. Further, the regression technique is usually used to identify the place of drowning by comparing the diatoms that have entered individual organs with the inhabitant diatoms in the water at the site of discovery [15–18]. Nevertheless, information obtained by such a method becomes gradually less reliable with progression of decomposition of the corpse; therefore, it may be inapplicable to cases that are discovered a long time after death. Accordingly, a method of estimating the time since death that is applicable to severely decomposed underwater corpses is desired.

Therefore, we attempted to develop a method of estimating the time since death in underwater corpses by applying forensic odontology techniques. We analyzed the changes over time in the types and quantities of seawater constituents deposited on dental enamel to determine the duration of underwater immersion in an attempt to estimate the time since death.

## Material and methods

### Analysis of seawater composition

One liter of seawater was collected in a coastal area and filtered, using Nalgene Rapid Flow Sterile Disposable Filter Units with PES Membrane (115 mL, 0.45 μm, pore) (Thermo Fisher Scientific, USA). After the filter paper was left to dry at room temperature, carbon was vapor-deposited using Carbon Coater (VC-100S, Vacuum Device, Japan). Then, seawater constituents were analyzed with the Electron Probe X-ray Micro Analyzer (EPMA; JXA-8200, JEOL, Japan).

**Fig. 1:**
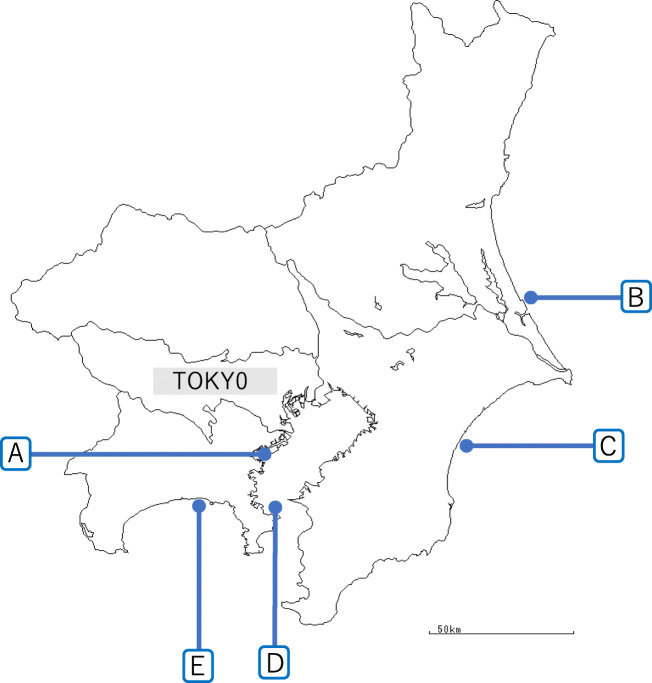
Water sampling points from the experimental area and surrounding seas. **a** Tokyo Bay (experimental area), **b** Kashima-Nada Sea, **c** Kujukuri Beach, **d** Uraga Channel, **e** Sagami Bay

The composition of seawater collected from Tokyo Bay from November 2016 to October 2017 was analyzed and classified by season, in order to confirm the changes in seawater composition during the year. At around the same time, seawater was collected from four different locations in Honshu, Japan (Kashima-Nada Sea, Kujukuri Beach, Uraga Channel, and Sagami Bay) for composition analysis by the same technique, aiming to study whether there was any difference in seawater composition between different areas (Fig. 1). The seawater used in this analysis was collected on the condition that no rain was recorded on the day or previous day. Seawater samples from Tokyo Bay, Kashima-Nada Sea, Kujukuri Beach, Uraga Channel, and Sagami Bay were classified as groups A, B, C, D, and E, respectively.

### Preparation of human dental specimens

Extracted human teeth that had been preserved in neutral formalin solution at our department were used in the present study. The teeth were rinsed with distilled water just before use. The teeth used in the study were randomly selected, regardless of age or sex. No pretreatment (cleaning, polishing) was conducted on the tooth surface prior to immersion.

After the human teeth were immersed in the collected seawater for a certain period, the specimens were left to stand in distilled water for 1 min to remove the salt crystals and left to dry at room temperature. The specimens were immersed for eight different periods: 0, 7, 14, 30, 60, 90, 180, and 210 days.

### Observation of deposits on tooth surface

After the specimens were left to stand in distilled water and then left to dry at room temperature, carbon vapor deposition was performed. Using a scanning electron microscope (SEM) (SU6600, HITACHI, Japan), observations were made of the deposits on the teeth surfaces.

### Composition analysis of the deposits on the teeth

Vapor deposition of carbon was conducted on the specimens, and quantitative analysis was made of the deposits on the enamel surface, using EPMA. Seawater composition was analyzed in advance, and O, S, Si, Cl, Na, Mg, Al, K, and Ca were detected as the main constituents. The study examined these main seawater constituents and two major dental components (Ca and P). Three specimens were prepared for each category. For each specimen, five measurement sites were randomly selected from the lip and cheek side. A total of 24 teeth were used for measurements in this study.

### Statistical analysis

Statistical analysis was performed using Welch’s *t* test and Kruskal-Wallis test. Regression analysis was conducted using Microsoft Excel software and Stat Plus LE.

### Ethical approval

The Medical and Ethical Committee in Tokyo Dental College approved all research protocols in this study, as well as the use of human samples (Approval Number: 715).

## Results

### Analysis of seawater constituents

**Fig. 2:**
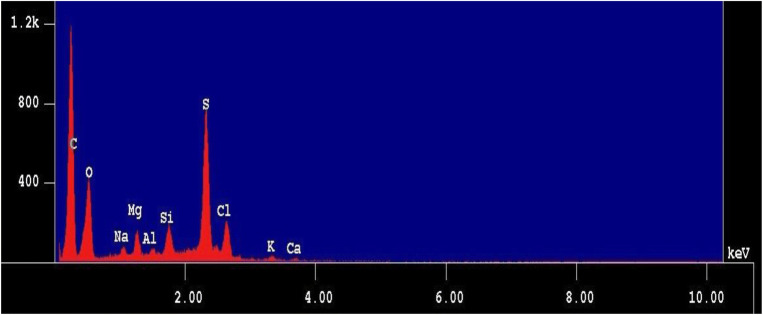
Constituents of seawater analyzed by electron probe micro analyzer. Mainly O, S, Si, Cl, Na, Al, Mg, K, and Ca were detected from seawater components. C was excluded because it was used for carbon vapor deposition

The following nine elements were mainly detected in the composition analysis of seawater using EPMA: O, S, Si, Cl, Na, Al, Mg, K, and Ca (Fig. 2). These nine elements were used for analysis of seawater composition in the present study. For analysis of the composition of the deposits on teeth, Na and Cl were excluded from the final study items as the water-washing process was added to remove the effect of salt crystals on other measurements.

### Changes in seawater composition during the year

**Fig. 3:**
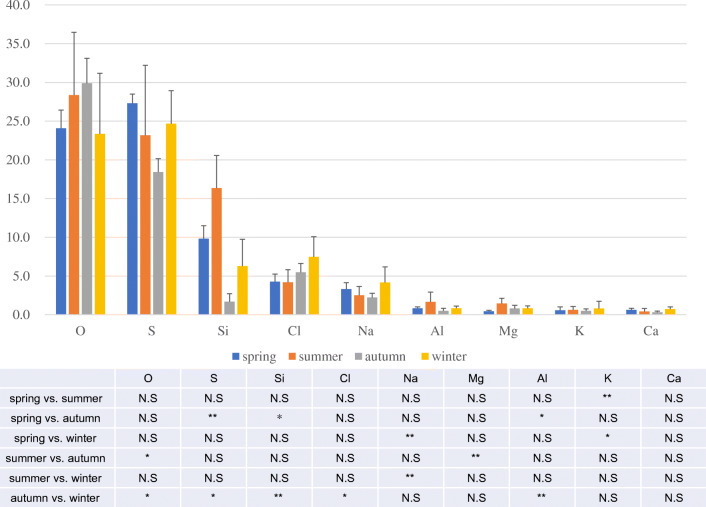
Transition of seawater component by season (spring: March to May, summer: June to August, autumn: September to November, winter: December to February). The upper stage shows the content of each component, and the lower stage shows the result of Kruskal-Wallis test. As a result of the Kruskal-Wallis test, there was a significant difference in a part of each component (*p < 0.05, **p < 0.01)

The composition of seawater that was collected monthly from the study areas differed significantly among seasons with respect to contents of some of the elements (spring: March–May; summer: June–August; autumn: September–November; winter: December–February). However, no significant seasonal patterns were observed with respect to element contents (Fig. 3).

### Differences in seawater composition between areas

**Fig. 4:**
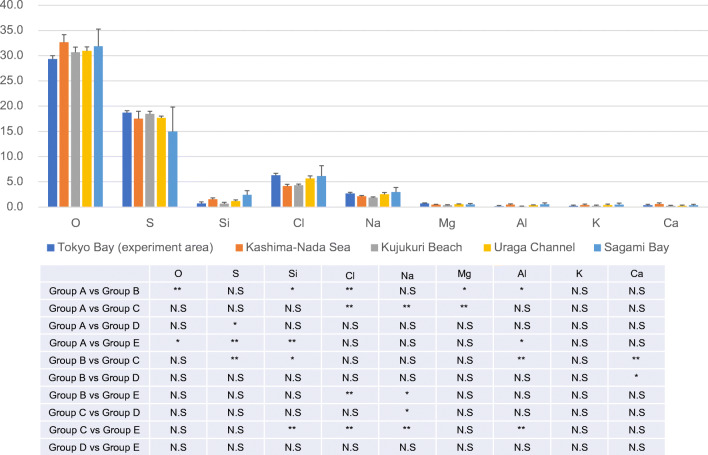
Difference in ingredients between experiment area and surrounding waters. The upper stage shows the difference in content of each component depending on the sea area, and the lower stage shows the result of Kruskal-Wallis test (group A: Tokyo Bay, group B: Kashima-Nada Sea, group C: Kujukuri Beach, group D: Uraga Channel, group E: Sagami Bay). As a result of the Kruskal-Wallis test, there was a significant difference in a part of each component (*p < 0.05, **p < 0.01)

In the present study, the seawater samples collected from the five study areas around the same time were compared with each other with respect to seawater composition. Although significant differences were observed in the contents of some of the elements among areas, no significant area-based patterns were observed with respect to element contents (Fig. 4).

### Observations of the deposits on the teeth surfaces using scanning electron microscope

**Fig. 5:**
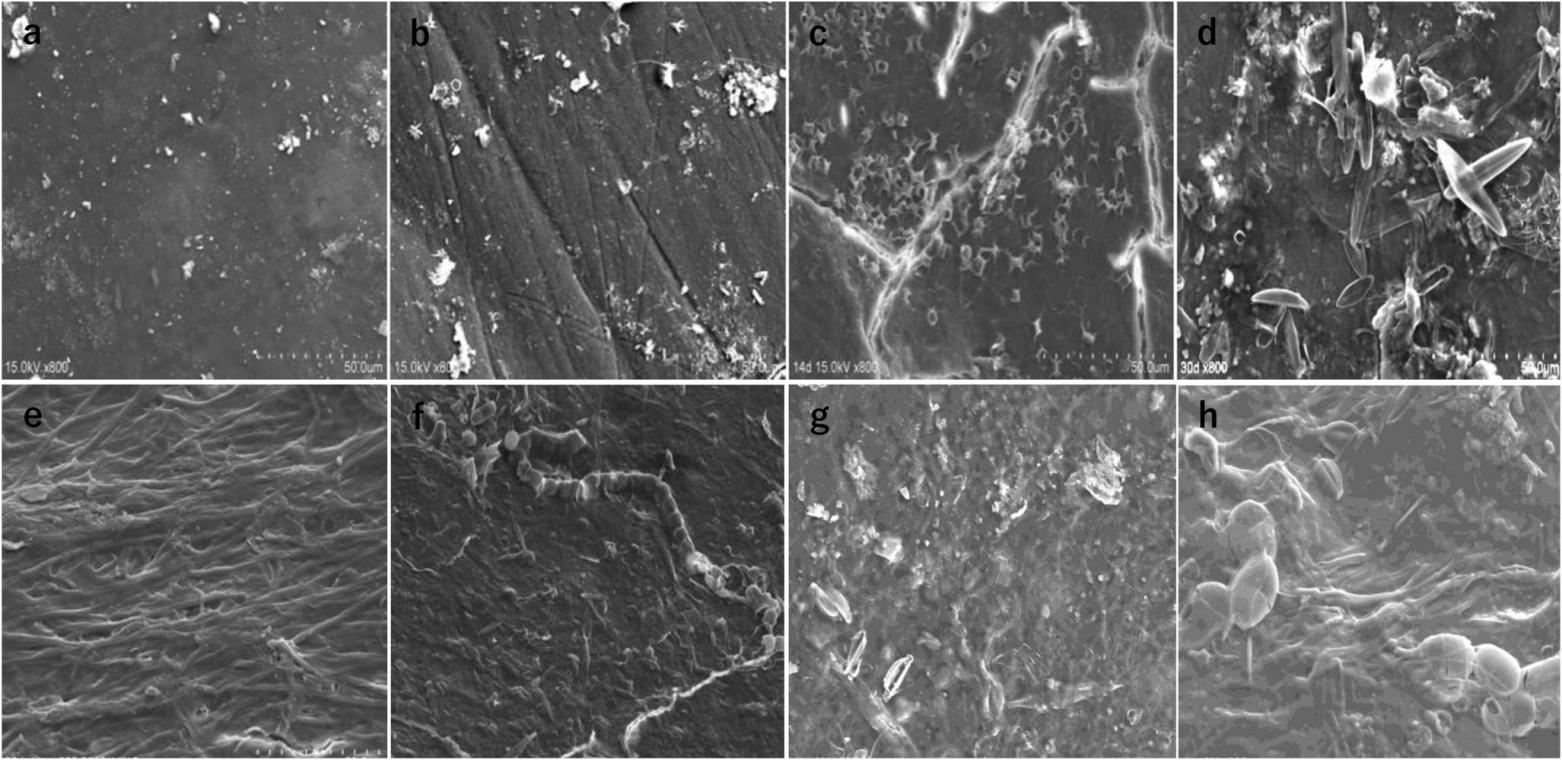
The surface of the enamel observed by immersion time using a scanning electron microscope (SEM). As the immersion time prolonged, the adhesion of phytoplankton to the enamel surface increased (**a** 0 day, **b** 7 days, **c** 14 days, **d** 30 days, **e** 60 days, **f** 90 days, **g** 180 days, **h** 210 days). All SEM images were taken at 800 power

Observations were made using the SEM images of the enamel surfaces of specimens, which had been classified into eight groups by duration of underwater immersion. The results confirmed that the quantity of vegetable plankton attached to the enamel surface increased with the extension of immersion time in water. Starting with the 14-day group, plankton attachment was observed where any color change of the enamel surface could not be identified by the naked eye, though the SEM image showed the attachment of vegetable plankton consisting mainly of diatoms distributed on the enamel surface. The amount of attached vegetable plankton further increased to cover a large part of the enamel surface in the 30-day group and the entire enamel surface in the 60-day and subsequent groups (Fig. 5).

### Analysis of composition of the deposits on the teeth surfaces

**Fig. 6:**
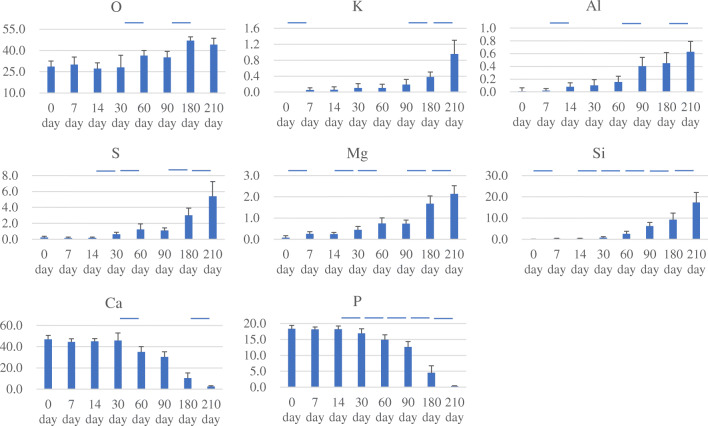
Composition analysis of deposits on the enamel surface. O, K, Al, S, Mg, and Si gradually increased and Ca and P decreased with prolongation of immersion time (*p < 0.01)

According to the EPMA analysis of the composition of the deposits on the enamel surfaces, highly significant differences were observed in the quantity of deposits on the enamel surfaces: between 30 and 60 days as well as 90 and 180 days for O; between 0 and 7 days, 14 and 30 days, 30 and 60 days, 60 and 90 days, 90 and 180 days, and 180 and 210 days for Si; between 0 and 7 days, 14 and 30 days, 30 and 60 days, 90 and 180 days, and 180 and 210 days for Mg; between 30 and 60 days and 180 and 210 days for Ca; between 14 and 30 days, 30 and 60 days, 60 and 90 days, 90 and 180 days, and 180 and 210 days for P; between 0 and 7 days, 90 and 180 days, and 180 and 210 days for K; between 7 and 14 days, 60 and 90 days, and 180 and 210 days for Al; between 14 and 30 days, 30 and 60 days, 90 and 180 days, and 180 and 210 days for S (*p* < 0.01) (Fig. 6).

The regression formula shown below was derived based on the above results (adjusted *R*^2^: 0.96).

$$ \mathrm{Immersion}\ \mathrm{period}\ \left(\mathrm{days}\right)=153.32449\hbox{--} 0.12318\times \mathrm{O}+0.10779\times \mathrm{S}\mathrm{i}+36.06503\times \mathrm{Mg}-0.53012\times \mathrm{Ca}-6.61794\times \mathrm{P}+16.35019\times \mathrm{K}+37.63482\times \mathrm{Al}-9.02016\times \mathrm{S}. $$

(The mass percentage of each element is to be assigned to each formula.)

## Discussion

In forensic medicine, estimation of time since death is as significant as diagnosis of cause of death. Postmortem phenomenon and plankton are usually examined for estimation of time since death, though the findings are insufficient for more detailed estimation in cases with lapses of long intervals after death. Plankton test is mainly used for diagnosis of drowning; however, seawater may enter various organs while floating. In the case of a long interval after death, determination of cause of death and estimation of time since death are more difficult [19–21]. Many of these techniques are inapplicable to a skeletonized body, in particular. Development of a method of estimating the time since death is desired especially for severely decomposed corpses including skeletonized bodies.

The present study was conducted from the perspective of forensic odontology. To date, the time since death has mainly been estimated based on the degree of decomposition. However, its drawback is low applicability to severely decomposed or skeletonized bodies. As a supplementary technique, our method can be applied in the case of a long interval after death. Dental enamel is the only hard tissue that is externally exposed even when the person is alive. The tooth itself is strongly fixed in the alveolar bone with a periodontal ligament, and therefore, is less likely to fall out, compared with nail or hair. Accordingly, analysis is possible even in the case of a long interval after death. The seawater components may also attach to bone after death. However, since the bone is surrounded by epithelial tissue and muscular tissue, the rate of decomposition of soft tissue should be considered in relation to the time required before the bone is exposed, lest the time since death may be greatly miscalculated. Moreover, similar considerations are required for evaluation of the cementum, including gingival atrophy and resorption of alveolar bone that already occurred in life. For these reasons, analysis of dental enamel is the most effective means to estimate the time since death, as it is not necessary to consider the condition of soft tissue.

Significant differences were readily observed in the quantity of some seawater component elements deposited on enamel, even after a short immersion time. With prolongation of immersion time, the differences increased markedly. Furthermore, when the immersion time was prolonged, more constituents showed significant differences. The quantity of the main components of enamel (Ca and P) that were detected decreased, while the quantity of deposits on the enamel surface increased. The possible reason was that electron beams radiated by EPMA reached the sample to a depth of about several micrometers and detected the corresponding characteristic X-ray. With the increase of the deposits on enamel surface, it possibly became more difficult to detect the components of enamel.

In addition, we examined the seasonal changes in seawater constituents. While no apparent regularity was found, significant differences were observed between seasons concerning certain constituents. However, it was difficult to classify the timing of drowning by season. Therefore, we may be able to derive a more exact formula for estimating the time since death, by increasing the number of constituents for analysis.

Furthermore, we analyzed the composition of the seawater obtained from regions other than the study area, aiming to find whether the regression formula obtained in the present study would be applicable to bodies found in other seas. The results were similar to those of seasonal analysis in the present study; namely, significant differences were observed only in certain constituents, although no clear difference was shown in the content ratio of each constituent. Accordingly, the finding suggested that the regression formula derived in the present study may be useful for other seas. In the present study, analysis was performed of minute amounts of the elements attached to the enamel surface. Therefore, careful attention should be paid while treating the specimens before analysis, to avoid excessive scraping or cleaning of tooth surface.

## Conclusion

In the present study, we analyzed the changes over time in the quantity of seawater constituents deposited on the enamel surface and derived an effective regression formula for estimating the time since death. Moreover, the technique of the present study can compensate for the drawback of the conventional method for estimating the time since death. Therefore, concurrent use of this method may increase the accuracy of estimation of the time since death.
